# ID 244 - Caderneta para a Pessoa Submetida ao Transplante Renal: uma proposta de tecnologia assistencial e educativa

**DOI:** 10.5327/2237-9622.2025.v34s1.244

**Published:** 2025-11-25

**Authors:** Dayani Galato, Samia Jucá Pinheiro, Klarissa Karine Lima Maracaipe, Jean Augusto da Silva, Carla Daniara Feitosa Coelho, Dayani Galato

**Keywords:** educação em saúde, prática clínica baseada em evidência, transplante de rim, tecnologia em saúde

## Abstract

**Introdução:**

Entre os transplantes de órgão sólidos, o transplante renal é o mais frequente no Brasil. Esse tratamento é considerado a melhor terapia substitutiva, seja com relação à melhora na qualidade de vida, seja pelos custos. Contudo, o manejo da saúde do paciente muda de forma importante após esse procedimento, pois é necessária a mudança tanto na terapia farmacológica, em especial com a inclusão de medicamentos imunossupressores, quanto no estilo de vida. Nesse sentido, esse trabalho tem como proposta apresentar o desenvolvimento de uma caderneta voltada ao acompanhamento da pessoa submetida ao transplante renal.

**Método:**

Trata-se de um estudo de desenvolvimento tecnológico usando métodos mistos. Para a identificação dos tópicos a serem abordados, foi realizada uma revisão integrativa da literatura, dois grupos focais com pessoas submetidas ao transplante renal e aplicação de questionários com profissionais vinculados à assistência dessas pessoas. A partir da lista identificada, foi desenvolvido o texto usando linguagem simples, bem como ilustrações para auxiliar a compreensão do texto. Esse projeto possui aprovação de um comitê de ética em pesquisa.

**Resultados:**

Na etapa da revisão, foram identificados 22 trabalhos que destacaram os temas infecções e rejeição do enxerto renal; comparecimento às consultas e aos exames; uso dos medicamentos; apoio nutricional; atividade física regular; sexualidade e planejamento familiar; proteção solar; apoio emocional e busca por estratégias de enfrentamento/ e apoio social. Com as pessoas e profissionais, emergiram dois grupos temáticos de tópicos: uso de medicamentos imunossupressores e hábitos alimentares no pós-transplante renal; e outros cuidados não farmacológicos e monitoramento de saúde no pós-transplante. A versão preliminar da caderneta tem 60 páginas, sendo 27 relacionadas à assistência e o restante à educação em saúde. Foram desenvolvidas mais de 90 ilustrações, buscando auxiliar a compreensão do texto de linguagem simples relacionado aos tópicos apresentados anteriormente. As ilustrações apresentam-se coloridas e inclusivas, ao trazer diversidade de personagens. A intenção é que a pessoa submetida ao transplante compreenda as informações e sinta-se contemplada no documento.

**Conclusão:**

Essa tecnologia se apresenta como uma proposta de ferramenta tanto assistencial como educativa voltada às pessoas submetidas ao transplante renal. Espera-se, nesse momento, aumentar a acessibilidade por meio da inclusão de QR Code para acesso à leitura destinado às pessoas com problemas de letramento ou acuidade visual, bem como garantir a aplicabilidade, por meio da análise por experts, como estratégia de construção das evidências de validação. Nesse sentido, espera-se que em breve ela possa ser aplicada em diversos serviços, vindo a atender milhares de pacientes.

